# Radiation-induced glymphatic dysfunction in patients with nasopharyngeal carcinoma: a study using diffusion tensor image analysis along the perivascular space

**DOI:** 10.3389/fnins.2023.1321365

**Published:** 2024-01-26

**Authors:** Xingyou Zheng, Jianchun Peng, Qing Zhao, Li Li, Jian-ming Gao, Keyang Zhou, Bei Tan, Lingling Deng, Youming Zhang

**Affiliations:** ^1^Department of Medical Imaging, The Fourth Hospital of Changsha, Changsha, Hunan, China; ^2^Department of Radiology, The Second Affiliated Hospital, Hengyang Medical School, University of South China, Hengyang, Hunan, China; ^3^Department of Radiology, Xiangya Hospital, Central South University, Changsha, China; ^4^National Clinical Research Center for Geriatric Diseases, Xiangya Hospital, Central South University, Changsha, Hunan, China; ^5^Sun Yat-sen University Cancer Center, State Key Laboratory of Oncology in South China, Collaborative Innovation Center for Cancer Medicine, Guangzhou, China; ^6^Department of Radiation Oncology, Sun Yat-sen University Cancer Center, State Key Laboratory of Oncology in South China, Collaborative Innovation Center for Cancer Medicine, Guangzhou, China

**Keywords:** radiation encephalopathy, glymphatic function, DTI-ALPS index, nasopharyngeal carcinoma, imaging biomarker radiation encephalopathy

## Abstract

Radiation encephalopathy (RE) refers to radiation-induced brain necrosis and is a life-threatening complication in patients with nasopharyngeal carcinoma (NPC) after radiotherapy (RT), and radiation-induced pre-symptomatic glymphatic alterations have not yet been investigated. We used diffusion tensor image analysis along the perivascular space (DTI-ALPS) index to examine the pre-symptomatic glymphatic alterations in NPC patients following RT. A total of 109 patients with NPC consisted of Pre-RT (*n* = 35) and Post-RT (*n* = 74) cohorts were included. The post-RT NPC patients, with normal-appearing brain structure at the time of MRI, were further divided into Post-RT-RE- (*n* = 58) and Post-RT-RE+ (*n* = 16) subgroups based on the detection of RE in follow-up. We observed lower DTI-ALPS _left_ index, DTI-ALPS _right_ index and DTI-ALPS _whole brain_ index in post-RT patients than that in pre-RT patients (*p* < 0.05). We further found that post-RT-RE+ patients demonstrated significantly lower DTI-ALPS _right_ (*p* = 0.013), DTI-ALPS _whole brain_ (*p* = 0.011) and marginally lower DTI-ALPS _left_ (*p* = 0.07) than Post-RT _non-RE_ patients. Significant negative correlations were observed between the maximum dosage of radiation-treatment (MDRT) and DTI-ALPS _left_ index (*p* = 0.003) as well as DTI-ALPS _whole brain_ index (*p* = 0.004). Receiver operating characteristic (ROC) curve analysis showed that DTI-ALPS _whole brain_ index exhibited good performance (AUC = 0.706) in identifying patients more likely developing RE. We concluded that glympathic function was impaired in NPC patients following RT and DTI-ALPS index may serve as a novel imaging biomarker for diagnosis of RE.

## Introduction

Nasopharyngeal carcinoma (NPC) is a malignancy of the nasopharynx cavity; it has a distinctive geographical distribution in east and Southeast Asia (Chen et al., 2019). Radiotherapy (RT) is one of the most important treatments for locally advanced NPC, which is highly sensitive to ionizing radiation (Kang et al., 2022). Although RT has led to great improvements in disease control and survival (Chua et al., 2016), radiation-related complications inevitably occur months, or even years, after RT treatment (Chen et al., 2019). Radiation-induced brain injury (RBI) is one of the most common radiation-induced complications. It has recently become a research focus for physicians because of its unclear pathological mechanisms and wide range of neuropsychiatric symptoms (such as cognitive decline and epilepsy) that worsen over time (Kang et al., 2022). RBI is pathophysiologically divided into three phases including acute reaction period (few days to few weeks), early delayed radiation period (1–6 months), and late delayed radiation period (6 months to few years) (Lin et al., 2017), while its clinical course is largely consisted of reversible (brain structures are normal-appearing after RT) and irreversible stages (MRI-detected brain necrosis, also known as radiation encephalopathy [RE]). Given that the progress of RBI into the irreversible phase leads to serious brain necrosis, more severe clinical symptoms and poor treatment outcomes, an investigation into the pathogenesis of RBI during the reversible phase is crucial for the early prevention of RE.

Many pre-symptomatic studies have reported that RBI is characterized by structural and functional alterations throughout the whole brain, rather than being confined to the temporal lobes only. For example, using advanced magnetic resonance imaging (MRI) sequences, several morphological and functional studies have reported altered gray matter volume, cortical thickness, local brain activity, and functional connectivity in brain regions not only within the temporal lobes (such as the inferior temporal gyrus and medial temporal lobe), but also outside the radiation field (such as the precentral gyrus and regions in the default mode network) (Lv et al., 2014; Lin et al., 2017; Ding et al., 2018; Zhang et al. 2020; Zhang W. et al., 2021). Moreover, numerous animal studies investigating the potential molecular substrates of RBI have reported that ionizing radiation-related inflammatory factors (including interleukin-1β, interferon-γ, and tumor necrosis factor-α) play an important role in blood–brain barrier (BBB) permeability, microvascular diameter, neuronal apoptosis, and glial proliferation (Raju et al., 2000; Yuan et al., 2003; Hwang et al., 2006; Wilson et al., 2009; Stielke et al., 2012; Yang et al., 2017), which in turn result in macroscopic structural and functional abnormalities. An excessive accumulation of inflammatory waste and the resulting neuroinflammatory pathology suggest that the removal of brain waste may be impeded (Lv et al., 2021). The glymphatic system—a perivascular network that exchanges brain metabolites between interstitial fluid and cerebrospinal fluid—is an emerging system responsible for the clearance of brain metabolic waste. Together with the well-known function of the glymphatic system, the presence of altered macroscopic brain structure and function and microscopic inflammatory waste accumulation in RBI suggests that an in-depth investigation into radiation-induced glymphatic system dysfunction may help our understanding of the potential neural mechanisms of this injury.

Diffusion tensor image analysis along the perivascular space (DTI-ALPS) is a non-invasive imaging method for quantitatively measuring the capacity of the glymphatic system (Taoka et al., 2017). This real-time method can evaluate glymphatic changes without using contrast agent, and is based on the knowledge that the direction of the perimedullary vein space is perpendicular to association and projection fibers (Taoka et al., 2017; Chen et al., 2021). Experimental evidence shows that DTI-ALPS findings are strongly correlated with results using the intrathecal method of glymphatic measurement, and a lower DTI-ALPS index is associated with poorer glymphatic clearance of waste products (Taoka and Naganawa, 2020; Shen et al., 2022). The DTI-ALPS index has thus been used to assess glymphatic system alterations in disorders such as neurodegenerative conditions, sleep disorders, and cerebrovascular disease (Chen et al., 2021; Lee et al., 2022; Zhang et al., 2022; Qin et al., 2023). However, few DTI-APLS studies have been conducted to investigate the alterations in activity of glymphatic system in RBI.

Given that neuroinflammatory pathology, vascular vulnerability, and the resulting deposits of neurotoxic agents in brain tissue have been reported after RT, we hypothesized that the glymphatic drainage system would be impaired in RT-treated patients with NPC. Furthermore, we predicted that these glymphatic impairments would be independent of radiation dose. To test our hypotheses, we first used DTI-APLS to measure glymphatic activity alterations in patients with NPC after RT, using DTI data. We then assessed the associations between the DTI-APLS index and radiation dose to confirm that glymphatic functional changes were likely induced by RT.

## Materials and methods

### Subjects

In the present study, 109 patients with NPC were included (74 in the post-RT group and 35 in the pre-RT group). The post-RT group was further divided into two subgroups based on the occurrence of RE in the follow-up (interval between RT and DTI examination <12 months): post-RT without RE (post-RT-RE−, *n* = 58) and post-RT with RE (post-RT-RE+, *n* = 16). Notably, NPC patients displayed normal-appearing brain morphology on the MRI scans at the beginning of our study. They were diagnosed with RE in the years of MRI follow-up after DTI examinations.

**Figure 1:**
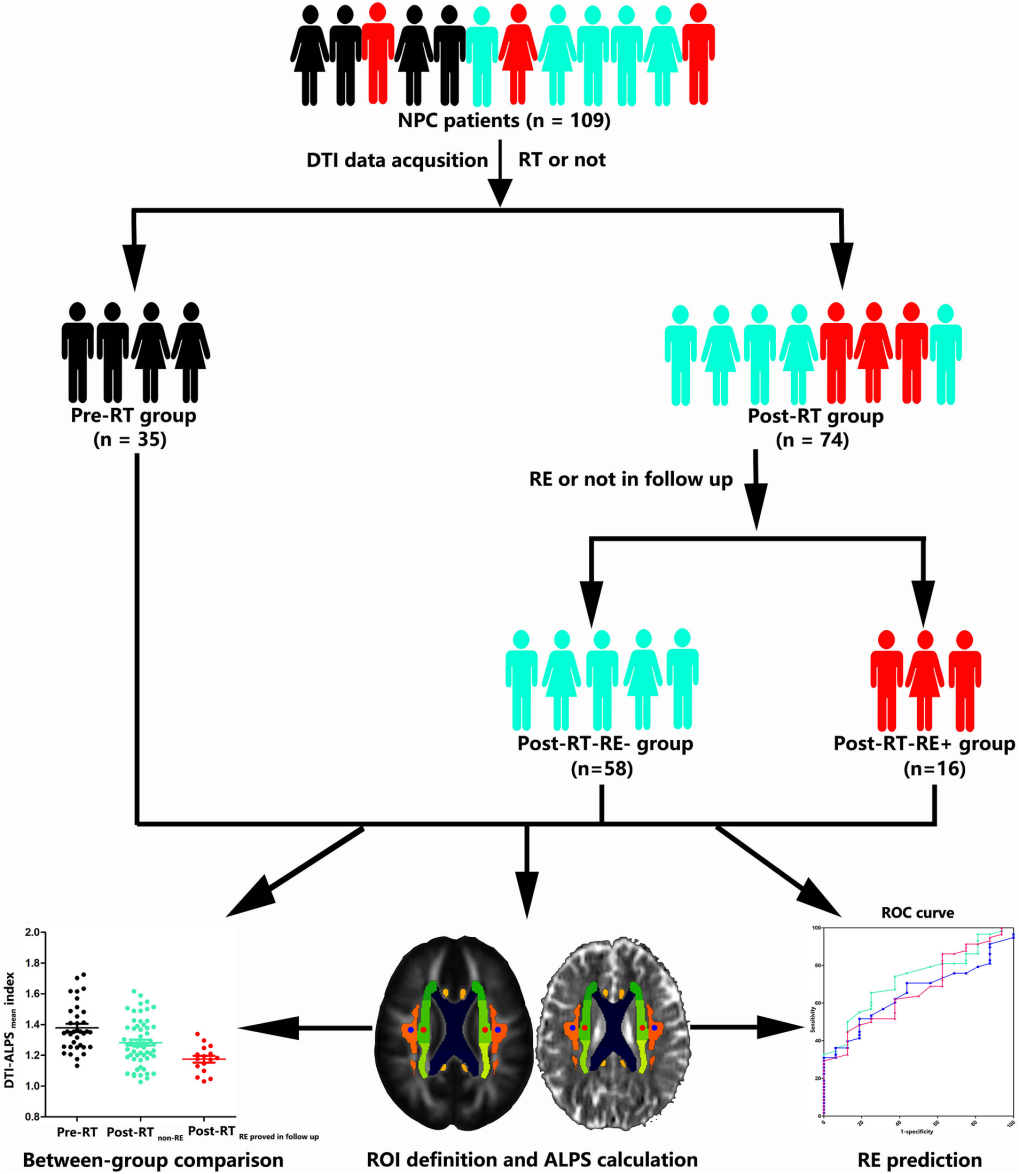
Skeleton diagram for grouping and analysis of the enrolled NPC patients. NPC, nasopharyngeal carcinoma; DTI, diffusion tensor imaging; RT, radiotherapy; RE, radiation encephalopathy; DTI-ALPS, diffusion tensor image analysis along the perivascular space; ROI, region of interest; ROC, receiver operator characteristic curve.

Figure 1 illustrates the whole technical route and main research contents of the present study. This study was approved by the Medical Research Ethics Committee of our hospital, and written informed consent was obtained from all subjects.

The RT and chemotherapy treatment information has been documented in our previous work (Zhang et al., 2018; Zhang Y. M. et al., 2021; Kang et al., 2022). In brief, intensity-modulated RT and two-dimensional conventional RT were applied to the post-RT patients. For patients staged IIb to IVa–b, concurrent chemoradiotherapy with/without neoadjuvant/adjuvant chemotherapy were administered. Detailed RT and chemotherapy information (Zhao et al., 2021) is provided in the Supplementary material. The 8th edition of the Union for International Cancer Control/American Joint Committee on Cancer Tumor, Node, Metastasis (TNM) system was used to determine the clinical stages of NPC lesions (Pan et al., 2016). Patients were enrolled if they met the following criteria: (1) pathologically confirmed NPC; (2) MRI detection of normal-appearing brain imaging; (3) right-handedness; (4) age < 68 years; and (5) Karnofsky Performance Status score > 80. Patients were excluded if they had: (1) MRI detection of brain invasion, orbital apex involvement or intracranial cranial nerve extension; (2) brain tumors or infectious disease (such as abscess); (3) history of head trauma or surgery; (4) history of neurological or psychiatric diseases; (5); severe brain atrophy (specific definition can be found in the supplementary materials); (6) contraindications (such as claustrophobia) for MRI scanning; (7) severe small vessel disease (deep and periventricular white matter hyperintensities, Fazekas score > 1); or (8) other substantial intracranial diseases (such as cerebral hemorrhage or stroke).

### MRI acquisition

MRI data were obtained on a Siemens Magnetom Tim Trio 3 T scanner. The DTI sequence was included in the present study for the subsequent DTI-ALPS data analysis. DTI data were collected transversely with an echo-planar imagingsequence using the following parameters: field of view = 256 mm × 256 mm, number of axial slices = 85, acquisition matrix = 128 × 128, slice thickness/gap = 2/0 mm, voxel size = 2 mm × 2 mm × 2 mm, repetition time = 10,800 ms, echo time = 87 ms, one image with b = 0 s/mm2, 30 images with b = 1,000 s/mm2, and number of excitations = 1.

### DTI data preprocessing

The main steps for DTI data preprocessing were as follows. First, we evaluated the artifacts (such as total geometric distortion, signal loss, and volume motion) and corrected the origin positions of all diffusion-weighted images. All diffusion-weighted image preprocessing was performed using FSL (FMRIB Software Library Package 6.0.1) (Smith et al., 2004).^1^ Non-brain voxels were extracted using BET and underwent motion correction and eddy current correction using FSL. The resulting data were then DTI fitted using FMRIB’s Diffusion toolbox to generate fractional anisotropy (FA) and mean diffusivity maps. Next, diffusivity maps of each subject were obtained using FSL software in the direction of the x-axis (right–left; Dxx), y-axis (anterior–posterior, Dyy), and z-axis (inferior–superior, Dzz).

### DTI-ALPS calculation

**Figure 2:**
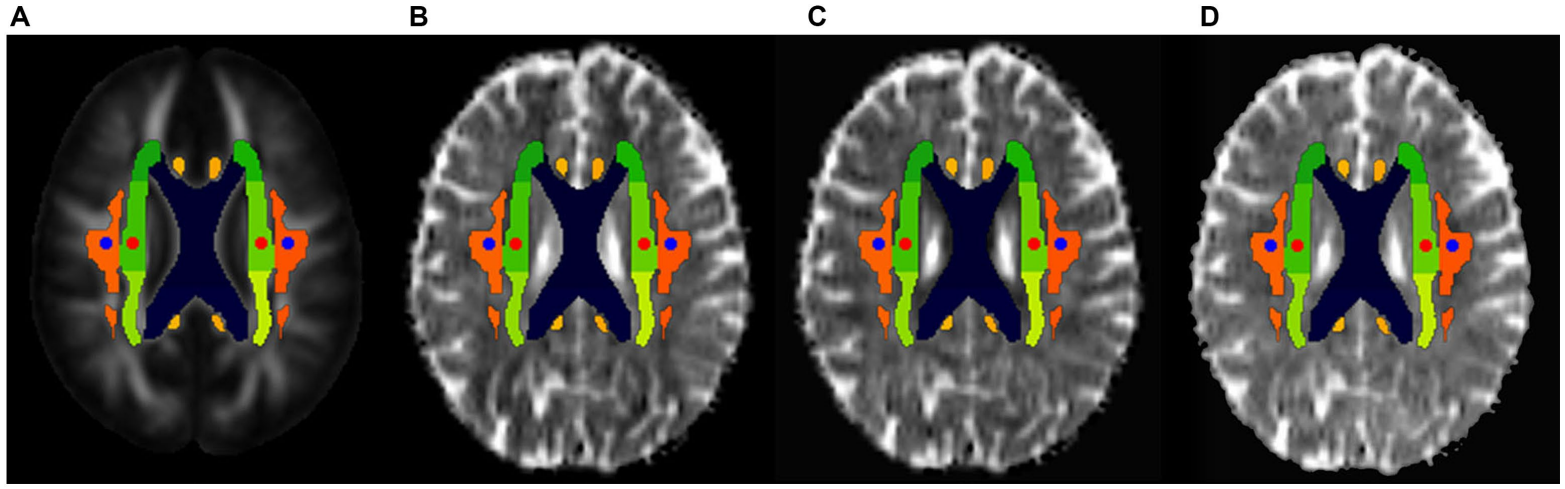
ROI selections for the DTI-ALPS index calculation. The placement of the ROIs (circles with a diameter of 5 mm) was determined on the color-coded fractional anisotropy (FA) maps, and these ROIs were placed in the bilateral projection fibers (blue circles) and association fibers (red circles) in FA atlas, respectively **(A)**. Then, these ROIs were placed on the diffusivity maps (spatially normalized) in the direction of the *x*-axis **(B)**, *y*-axis **(C)**, and *z*-axis **(D)**.


The DTI-ALPS index is widely used in neurological disorders, including brain injury. An improved ALPS calculation method (Zhang W. et al., 2021) was used to calculate the ALPS index. Specifically, we first registered the diffusion maps obtained in the x-axis (right–left; Dxx), y-axis (anterior–posterior, Dyy), and z-axis (inferior–superior, Dzz) directions into the standard FA map template of the ICBM-DTI-81 Atlas.^2^ Previous studies have generally used susceptibility-weighted imaging to assist in selecting the position of the uppermost layer of the lateral ventricle body perpendicular to the medullary vein, to obtain different diffusion coefficients to calculate the ALPS. However, because most patients have the same direction of medullary veins in the uppermost layer of the lateral ventricle body, regions of interest (ROIs) can be placed using DTI images only, without relying on susceptibility-weighted imaging. Using a standard color-coded FA map, spherical ROIs measuring 5 mm in diameter were placed in the projection and association areas at the level of the bilateral lateral ventricle body (Figure 2). The ROI positions were visually confirmed for each patient. If needed, manual corrections were performed by slightly moving the ROIs. Subsequent extractions of the diffusion coefficients of these ROIs on the x-, y-, and z-axes were performed in the subsequent analysis. In the projection fiber area, the main fibers run along the z-axis direction, while the x-and y-axes are perpendicular to the main fibers. By contrast, in the association fiber area, the main fibers run in the direction of the y-axis, and the x-and z-axes are perpendicular to the main fibers. The ALPS index was therefore derived from the ratio of the average values of x-axis diffusivity in the projection fiber area (Dx_proj) and the x-axis diffusivity in the association fiber area (Dx_assoc) to the average values of the y-axis diffusivity in the projection fiber area (Dy_proj) and the z-axis diffusivity in the association fiber area (Dz_assoc), as follows:

$$\mathrm{ALPS}-\mathrm{index}=\frac{\mathrm{meanDx}:\mathrm{proj},Dx:\mathrm{assoc}}{\mathrm{meanDy}:\mathrm{proj},Dz:\mathrm{assoc}}$$

In the present study, the left, right, and the average ALPS index values across hemispheres (DTI-ALPS_wholebrain_) were evaluated separately.

## Statistical analysis

### Clinical data analysis

We used a number of statistics to depict different data types. Specifically, we used the mean and standard deviation to describe quantitative clinical data with normal distribution; the median and interquartile range were reported when clinical data were non-normally distributed. Qualitative clinical data were presented using frequencies. We compared intergroup differences in clinical stage, sex, and RT technique using the chi-squared test, and compared intergroup differences in age using one-way analysis of variance. For the post-RT subgroups, we compared intergroup differences in maximum temporal lobe RT dosage and the interval between RT and DTI examination using a two-sample *t*-test. For all analyses, *p* < 0.05 was considered significant.

### DTI-ALPS analysis

Intergroup differences in the DTI-ALPS index were compared at three levels: the DTI-ALPS index in the left hemisphere (DTI-ALPS_left_), the DTI-ALPS index in the right hemisphere (DTI-ALPS_right_), and the average DTI-ALPS index across hemispheres (DTI-ALPS_wholebrain_). We compared the intergroup differences in DTI-ALPS_left_, DTI-ALPS_right_, and DTI-ALPS_wholebrain_ using one-way analysis of variance. *Post hoc* tests were performed using the least significant difference method. For all analyses, *p* < 0.05 was considered significant.

A correlation analysis was used to explore the relationship between the DTI-ALPS index (including DTI-ALPS_left_, DTI-ALPS_right_, and DTI-ALPS_wholebrain_) and the ipsilateral maximum dosage of radiation-treatment (MDRT); *p* < 0.05 was considered significant.

A receiver operating characteristic (ROC) curve was used to assess the RE diagnostic performance of the DTI-ALPS index. In terms of diagnostic performance, area under the curve (AUC) values between 0.9 and 1.0 are considered excellent, values between 0.7 and 0.9 are considered good, and values between 0.6 and 0.7 are considered fair (El Khouli et al., 2009). The cut-off value was equal to the maximum of Youden’s index, which was calculated as (sensitivity + specificity) − 1.

## Results

### Clinical parameters

This study consisted of 86 male and 23 female patients with NPC. Ages ranged from 22 to 67 (mean 45.61) years. Tumor stage ranged from T1N1M0 to T4N3M0 in the pre-RT and post-RT-RE− groups, and from T1N0M0 to T4N2M0 in the post-RT-RE+ group. No significant differences were observed between the pre-RT, post-RT-RE−, and post-RT-RE+ groups in age (*p* = 0.091), sex (*p* = 0.211), or clinical stage (*p* = 0.304). In the post-RT group, there were no significant differences between the post-RT-RE− and post-RT-RE+ subgroups in the time interval between RT and DTI examination (*p* = 0.278), RT technique (*p* = 0.955), or MDRT to the left (*p* = 0.744) or right (*p* = 0.683) temporal lobes. In the post-RT-RE+ group, brain necrotic lesions in the left, right, and bilateral temporal lobes were observed in seven, four, and five patients, respectively (Table 1). The time interval between DTI examination and the diagnosis of RE is 34.37 ± 14.68 months.

**Table 1:** Demographic and clinical data of patients with NPC.

Clinical features	Pre-RT group (*n* = 35)	Post-RT-RE-group (*n* = 58)	Post-RT-RE+ group (*n* = 16)	*p* value
*Age (years) mean ± SD*	46.71 ± 9.02	43.95 ± 8.43	49.19 ± 10.04	0.079
*Sex, n*				
Male	26	45	15	0.269
Female	9	13	1	
*Clinical staging #*				
I/II, *n*	3^a^	7^b^	4^c^	0.240
III/IV, *n*	25^a^	42^b^	9^c^	
*Cervical lymphatic involvement*				
*N0*	4^a^	2^b^	1^c^	0.290
*N1-3*	24^a^	47^b^	13^c^	
*Time intervals between RT and DTI examinations (month)*	NA	6.81 ± 4.47	9.00 ± 9.81	0.397
*RT technology*				
IMRT, *n*	NA	48	13	0.888
others, *n*	NA	10	3	
*Chemotherapy or not*				
No	NA	2^b^	1^c^	0.662
Yes	NA	46^b^	13^c^	
*Maximum dosage of RT for temporal lobes (Gy)*				
Left	NA	66.91 ± 7.56^#^	68.89 ± 6.79^*^	0.268
Right	NA	66.84 ± 7.59^#^	67.82 ± 10.26^*^	0.789
*The location of RE*				
Right, *n*	NA	NA	4 (25.00)	NA
Left, *n*	NA	NA	7 (43.75)	
Bilateral, *n*	NA	NA	5 (31.25)	

NA denotes not available; a–c denote 7, 9, and 3 patients’ clinical stageing were not available from medical records; #denotes radiation dose of 35 patients were available; *denotes radiation dose of 7 patients were available.

### DTI-ALPS index

**Figure 3:**
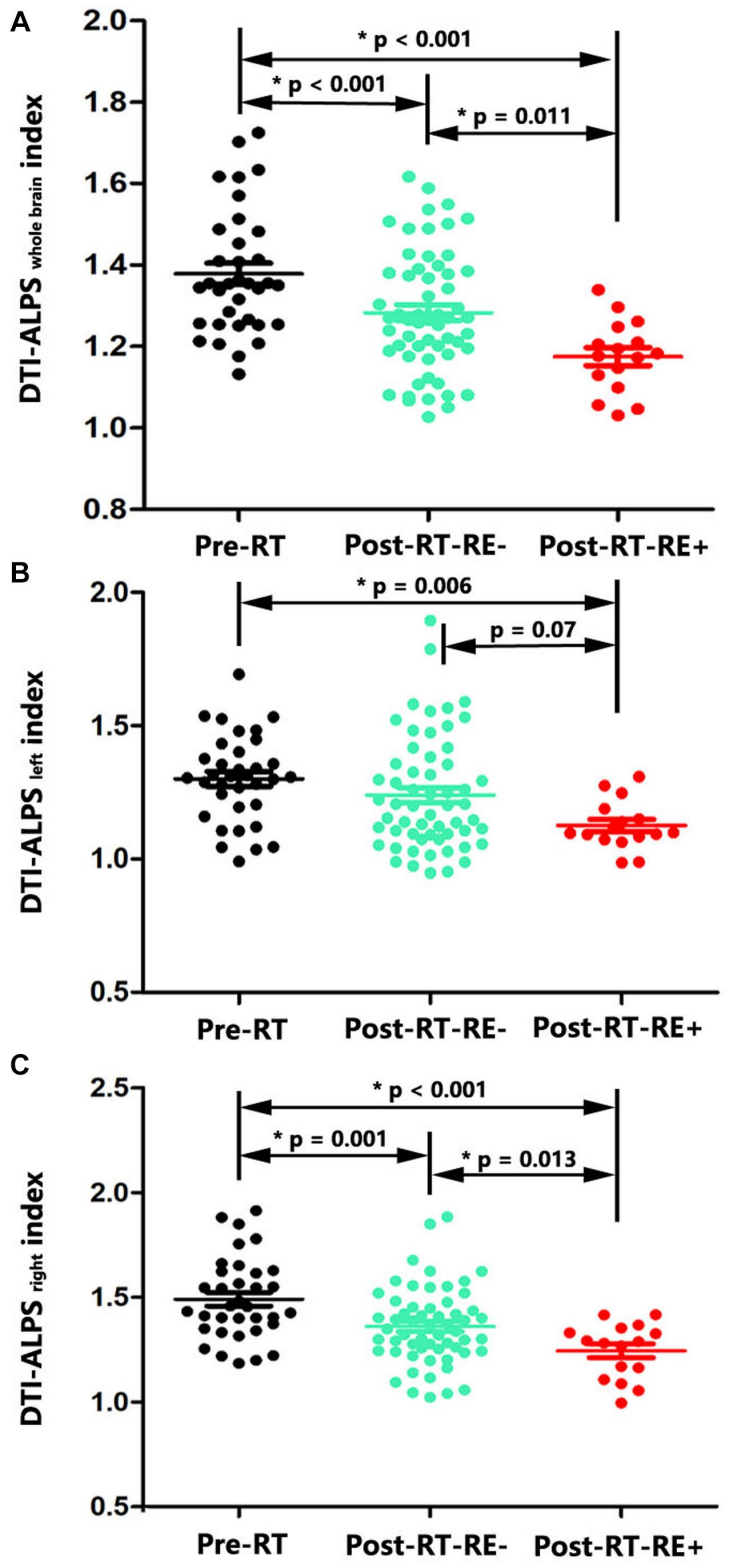
Between-group differences in DTI-ALPS index. Significant reductions of DTI-ALPS _whole brain_ index were observed in the pairwise comparisons of Post-RT-RE+ vs. Pre-RT (*p* < 0.001), Post-RT-RE-vs. Pre-RT (*p* < 0.001), and Post-RT-RE+ vs. Post-RT-RE- (*p* = 0.011) **(A)**. Compared with the Pre-RT group, patients in Post-RT-RE+ displayed significant reduction of DTI-ALPS _left_ index (*p* = 0.006). Compared with the Post-RT-RE-group, patients in Post-RT-RE+ group showed marginally significant reduction of DTI-ALPS _left_ index (*p* = 0.07) **(B)**. Significant reductions of DTI-ALPS _right_ index were observed in the pairwise comparisons of Post-RT-RE+ vs. Pre-RT (*p* < 0.001), Post-RT-RE-vs. Pre-RT (*p* = 0.001), and Post-RT-RE+ vs. Post-RT-RE- (*p* = 0.013) **(C)**.

For DTI-ALPS_wholebrain_, significant intergroup differences were observed between the pre-RT, post-RT-RE−, and post-RT-RE+ groups (*p* < 0.001). Subsequent *post hoc* testing revealed that DTI-ALPS_wholebrain_ was significantly lower in the post-RT-RE+ group than in the pre-RT (*p* < 0.001) and post-RT-RE− (*p* = 0.011) groups. Moreover, DTI-ALPS_wholebrain_ was significantly lower in the post-RT-RE− group than in the pre-RT group (*p* = 0.002) (Figure 3A).

For DTI-ALPS_left_, significant intergroup differences were observed between the pre-RT, post-RT-RE−, and post-RT-RE+ groups (*p* = 0.021). Subsequent *post hoc* testing revealed that DTI-ALPS_left_ was significantly lower in the post-RT-RE+ group than in the pre-RT group (*p* = 0.006). Furthermore, a marginally significant difference in DTI-ALPS_left_ was observed between the post-RT-RE+ and post-RT-RE− groups (*p* = 0.07). No significant differences were observed between the pre-RT and post-RT-RE− groups (*p* = 0.133) (Figure 3B).

For DTI-ALPS_right_, significant intergroup differences were observed between the pre-RT, post-RT-RE−, and post-RT-RE+ groups (*p* < 0.001). Subsequent *post hoc* testing revealed that DTI-ALP_right_ was significantly lower in the post-RT-RE+ group than in the pre-RT (*p* < 0.001) and post-RT-RE− (*p* = 0.013) groups. Moreover, DTI-ALPS_right_ was significantly lower in the post-RT-RE− group than in the pre-RT group (*p* = 0.001) (Figure 3C).

### Correlation analysis

In the post-RT group, there was a significant negative correlation between DTI-ALPS_left_ and ipsilateral MDRT (*r* = −0.472, 95% confidence interval [−0.688, −0.179], *p* = 0.003). There was also a significant negative correlation between DTI-ALPS_wholebrain_ and left-side MDRT (*r* = −0.460, 95% confidence interval [−0.680, −0.164], *p* = 0.004). No significant correlations were observed between DTI-ALPS_right_ or DTI-ALPS_wholebrain_ and right-side MDRT (*p* = 0.854 and 0.150, respectively).

### ROC analysis

**Figure 4:**
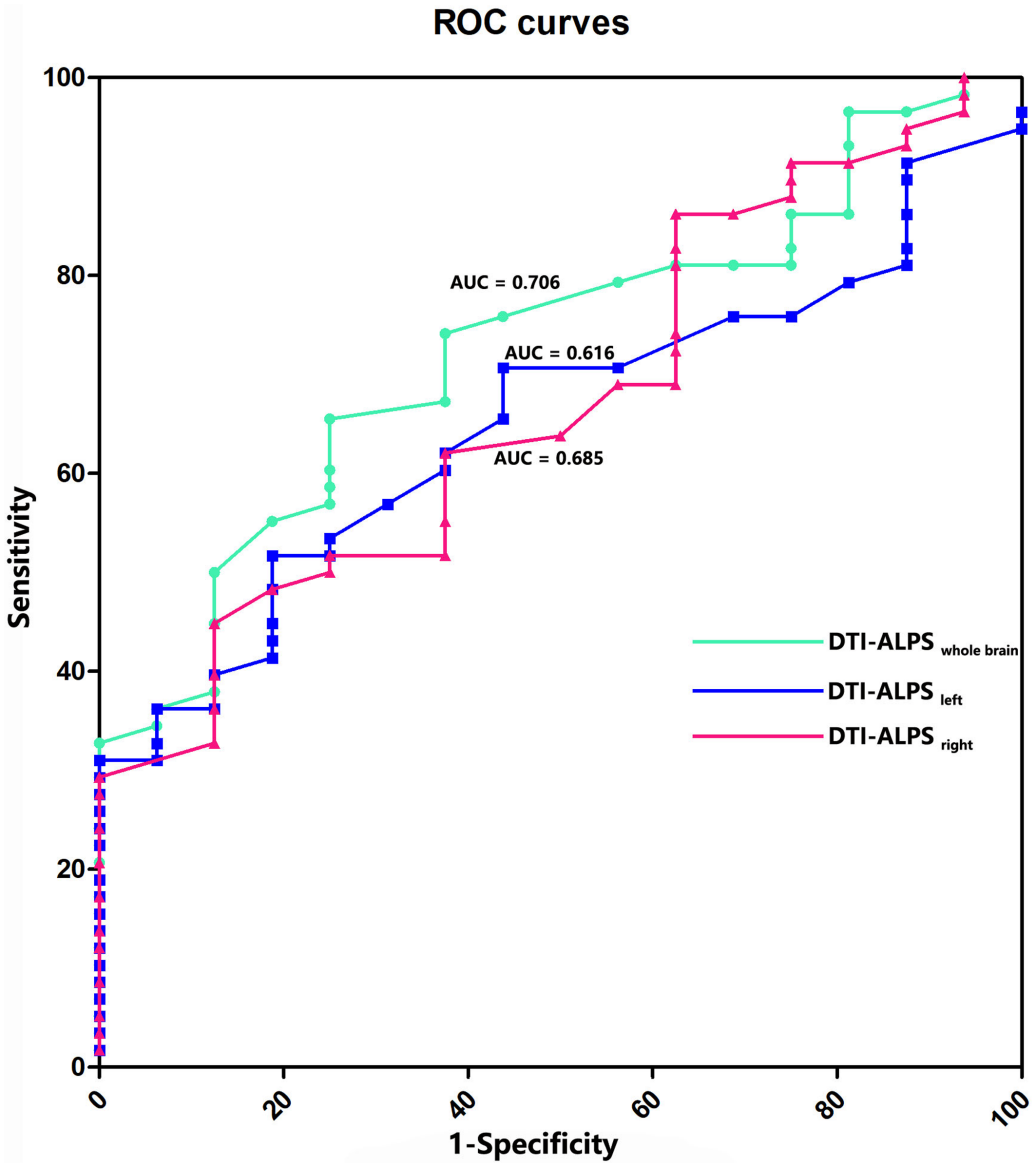
ROC curves for prediction of RE. The AUC value is 0.706 for the whole brain ALPS index (*p* = 0.009), 0.616 for the left ALPS index (*p* = 0.139), and 0.685 for the right ALPS index (*p* = 0.018).

The mean, left, and right ALPS indexes were used to evaluate the diagnostic performance of RE. The AUC value was 0.706 for the mean ALPS index (*p* = 0.009), 0.616 for the left ALPS index (*p* = 0.139), and 0.685 for the right ALPS index (*p* = 0.018). The AUC value of the ALPS_wholebrain_ index was the highest, with a cut-off value of 1.22, sensitivity of 67.2%, and specificity of 72.2% (Figure 4).

## Discussion

To our knowledge, the present report details the first attempt to use the DTI-ALPS index to examine the effects of RT on the glymphatic clearance system in patients with NPC. Patients with NPC in the post-RT group (comprising both the post-RT-RE− and post-RT-RE+ subgroups) had a significantly lower DTI-ALPS index than those in the pre-RT group, suggesting that glymphatic function is impaired in NPC patients following RT. Notably, patients with NPC in the post-RT-RE+ subgroup had significantly lower DTI-ALPS_right_ and DTI-ALPS_wholebrain_ values and marginally lower DTI-ALPS_left_ values than post-RT-RE− patients; further ROC analysis revealed that the DTI-ALPS_wholebrain_ index was able to effectively differentiate NPC patients who developed RE in the follow-up from those who did not. These findings suggest that the DTI-ALPS index may serve as a potential biomarker for the early diagnosis of RE. In addition, the significant correlations between the MDRT and the DTI-ALPS index indicate that the insufficient clearance system function was likely induced by RT. Together, these findings may contribute to a better understanding of the underlying neural mechanisms of RE.

Compared with patients in the pre-RT group, those in the post-RT group had a significantly lower DTI-ALPS index. This finding is consistent with that of a recent diffusion-weighted imaging study in which the diffusion-weighted imaging-ALPS index was significantly lower in the post-radiation group than in the control group (Taoka et al., 2023). A decreased DTI-ALPS index may thus indicate glymphatic system insufficiency in patients with NPC following RT. Anatomically and physiologically, the glymphatic system runs parallel to central nervous system vasculature in a perivascular space that is enclosed by astrocytic endfeet; it mediates substrate exchange between cerebrospinal fluid and interstitial fluid (ISF) to remove extracellular waste (Radford et al., 2015; Plog and Nedergaard, 2018; Hablitz and Nedergaard, 2021). Any pathophysiological processes or molecular events that affect structural components of the glymphatic system can result in the decreased removal of neurotoxic metabolites (Iliff et al., 2013; Radford et al., 2015; Mogensen et al., 2021; Wei et al., 2023). Interestingly, the pathophysiological hallmark of RBI is tightly linked to an altered tissue microenvironment consisting of overloaded inflammatory factors (such as tumor necrosis factor-α, cell adhesion molecules, and cytokines), BBB disruption, glial cell apoptosis, and varying degrees of vascular damage (Raju et al., 2000; Yuan et al., 2003; Wilson et al., 2009; Yang et al., 2017). These factors are related to pathological reactions, such as the perivascular accumulation of superfluous inflammatory substances, endothelial apoptosis-related BBB leakage, and vascular defect-induced pulse pressure disruption, and are reportedly the key contributors to insufficient glymphatic clearance (Kress et al., 2014; Verheggen et al., 2018; Ekenze et al., 2023; Kern et al., 2023). Together with the observation that pathogenic factors are interrelated in RBI, it is therefore tempting to speculate that the impaired glymphatic function in post-RT patients in the present study may be the result of multiple superimposed insults from diverse molecular events or pathological processes.

Our findings of the lowest DTI-ALPS index in post-RT-RE+ patients, and of its impressive predictive performance for RE, are of particular interest. Such findings indicate that the DTI-ALPS index may serve as a potential biomarker for the early diagnosis of RE/brain necrosis (if glymphatic function worsens) in post-radiation NPC patients. Early RBI may be regarded as a pathological state characterized by a dynamic balance of damage and repair; brain necrosis can occur when this balance is disrupted (Steel et al., 1986; Yang et al., 2017). Findings from numerous animal models indicate that self-healing systems can be activated immediately after RT to protect against neurological deterioration through the inhibition or reduction of RT-related oxidative stress and apoptosis (Wang et al., 2016; Soria et al., 2019). As a self-healing system, the glymphatic system may exert its neuroprotective effects on damaged brain tissue by enhancing its drainage function, to remove metabolic neurotoxic products (Dai et al., 2023). Such a role may also be supported by a study of a healthy population showing that increased glymphatic clearance, driven by physical activity, plays an essential role in maintaining a healthy brain throughout aging (Vecchio et al., 2018). It is thus reasonable to speculate that progressive glymphatic impairments might be the key drivers for the phenoconversion from reversible RBI to clinically irreversible radiation-induced brain necrosis/RE. However, the probable reasons for our observation of the most severe glymphatic impairments in post-RT-RE+ patients remain unknown and require further investigation. Nonetheless, the glymphatic system may be a novel therapeutic target for alleviating the neuropsychiatric symptoms of RE.

Our findings of significant negative correlations between the MDRT and the DTI-ALPS index indicate that a higher ipsilateral maximum RT dosage is associated with a lower DTI-ALPS index. This expected finding implies that glymphatic functional impairments are mainly induced by RT, and is in line with one recent whole-brain radiation study showing that the ALPS index has a weak negative correlation with biologically equivalent RT doses (Taoka et al., 2023). Moreover, using rat models, several studies have reported that increased single or total radiation doses result in elevated inflammatory factors (such as tumor necrosis factor-α, interferon-γ, and interleukin-1β), cellular debris, and BBB permeability, and have a negative effect on perivascular astrocyte survival (Chiang et al., 1993; Raju et al., 2000; Yuan et al., 2003; Yang et al., 2017). Coincidentally, as reported in previous studies, these RT-related pathological events can in turn aggravate glymphatic system dysfunction through possible mechanisms that include inadequate competence in dealing with overloaded waste, turbulent convective flux through the interstitium along the central nervous system vasculature, and aquaporin-4 depolarization in damaged astrocytic endfeet (Kress et al., 2014; Jessen et al., 2015; Radford et al., 2015). The proposed hypothesis, that the observed glymphatic dysfunction is mainly driven by ionizing radiation exposure in patients with NPC, is therefore both etiologically and pathologically reasonable.

Several limitations of the present study should be mentioned. First, the DTI-ALPS index cannot assess whole-brain glymphatic function because it is based on the orthogonal geometric relationship between projection and association fibers and medullary arteries and veins in the lateral ventricle body (Taoka et al., 2017; Kamagata et al., 2022). The DTI-ALPS index thus warrants cautious interpretation and further investigation. Nevertheless, considering that RBI can occur not only in the temporal lobe, but also in almost all areas of the brain, the DTI-ALPS index measured at the lateral ventricle level may reflect—at least to some extent—important RT-related pathological processes. Second, several confounding factors (such as chemotherapeutic agents, varying radiation fields and potential small vessel disease) were present in the present study. Because their negative confounding effects were unable to be completely eliminated, the reliability of our results may be compromised. Third, the lack of sufficient biological and cognitive data weakens the interpretability of our findings. Fourth, a relatively small sample size, such as that of the current study, may lower the statistical power and result in false-negative error, which then lowers our confidence to reach a definitive conclusion. Fifth, although the between-group difference of cervical lymphatic metastases in post-RT NPC patients were not significant, their potential confounding effects on glymphatic changes could not be ruled out. Finally, the cross-sectional study design impeded investigations into temporal alterations in the glymphatic clearance network at different stages of RBI. Given the aforementioned limitations of our study, future longitudinal investigations with large sample sizes and comprehensive multimodal data are warranted to further validate our findings.

## Conclusion

A radiation-induced reduction in the DTI-ALPS—a potential biomarker for the early diagnosis of RE—was observed in patients with NPC, indicating that glymphatic dysfunction may contribute to the pathogenesis of RBI.

## Data availability statement

The original contributions presented in the study are included in the article/Supplementary material, further inquiries can be directed to the corresponding author.

## Ethics statement

The studies involving humans were approved by Medical Research Ethics Committee of Xiangya hospital, Central South University. The studies were conducted in accordance with the local legislation and institutional requirements. The participants provided their written informed consent to participate in this study.

## Author contributions

XZ: Data curation, Formal analysis, Methodology, Writing – original draft. JP: Software, Writing – original draft. QZ: Investigation, Conceptualization. LL: Conceptualization, Supervision, Writing – review & editing. J-mG: Data curation, Supervision, Writing – review & editing. KZ: Investigation, Writing – review & editing. BT: Investigation, Writing – review & editing. LD: Formal analysis, Investigation, Writing – review & editing. YZ: Conceptualization, Data curation, Funding acquisition, Investigation, Supervision, Writing – review & editing.

## Footnotes

^1^ http://www.fmrib.ox.ac.uk/fsl

^2^ https://www.loni.usc.edu/research/atlases
